# Transcriptional accuracy modeling suggests two-step proofreading by RNA polymerase

**DOI:** 10.1093/nar/gkx849

**Published:** 2017-09-28

**Authors:** Harriet Mellenius, Måns Ehrenberg

**Affiliations:** Department of Cell and Molecular Biology, Uppsala University, Uppsala 752 37, Sweden

## Abstract

We suggest a novel two-step proofreading mechanism with two sequential rounds of proofreading selection in mRNA transcription. It is based on the previous experimental observations that the proofreading RNA polymerase cleaves off transcript fragments of at least 2 nt and that transcript elongation after a nucleotide misincorporation is anomalously slow. Taking these results into account, we extend the description of the accuracy of template guided nucleotide selection beyond previous models of RNA polymerase-dependent DNA transcription. The model derives the accuracy of initial and proofreading base selection from experimentally estimated nearest-neighbor parameters. It is also used to estimate the small accuracy enhancement of polymerase revisiting of previous positions following transcript cleavage.

## INTRODUCTION

The accuracy of an enzymatic reaction reflects the enzyme’s preference of a correct substrate over other substrates in product formation. Genetic information transmitting reaction systems like transcription, translation and replication require particularly high accuracy, since a flawless product from any of these processes depends on a chain of multiple elongations events, all of which must be correct. In *Escherichia coli*, an error free transcript of a typical gene needs 1000 correct transcript elongations in a row. Accordingly, the accuracy of transcription has evolved to an average nucleotide misincorporation error frequency in the 10^-5^–10^−4^ range (1–4), leading to a flawless transcript probability in the 99–90% range.

A further challenge in transcription is that the same polymerase structure must probe several types of substrates, which are correct or incorrect only depending on the DNA template in the active site. The free energy difference, *ΔG^‡,nc^ - ΔG^‡,c^*, between the standard free energy of template binding for a cognate and a non-cognate nucleotide in the catalytic site of the polymerase determines the accuracy of substrate selection. At the same time, the catalytic site may be designed for maximal accuracy of substrate selection by favoring Watson–Crick geometry for the substrate–template nucleotide pair (5) and water exclusion to magnify the selective impact of inter-nucleotide hydrogen bonds (6). Finally, the transcription elongation reactions also have to be fast for quick assembly of the long reaction product chains, in spite of the universal rate–accuracy trade-off in enzymatic reactions (7). There is strong experimental evidence for transcriptional accuracy amplification by proofreading through transcript cleavage after polymerase backtracking (8). However, precise experimental estimates of transcriptional accuracy and the contributions from initial and proofreading selection have remained hard to come by (2,9).

We previously suggested that the accuracy of nucleotide selection varies by several orders of magnitude in a DNA template dependent manner (10). Our modeling approach is primarily based on experimentally determined standard free energies of melting of the double stranded DNA and the RNA/DNA hybrid in the transcription bubble. This melting energy varies for all combinations of pairs of base pairs due to the effect of base stacking on the base pair interaction (11–13). As the movement of the polymerase and the other reactions in the nucleotide addition cycle induce alterations to the transcription bubble, forming or breaking base pairs, the unique free energy of the sequence in the transcription bubble determines the reaction rate constants in transcription. The accuracy variation is hence due to the movement of the transcription complex through a rugged*-*free energy landscape along the template sequence, shaped by the ever varying free energy of interactions between pairs of base pairs. The advantage of this approach is that we can make pertinent predictions about how transcriptional accuracy varies along known template sequences.

**Figure 1.**
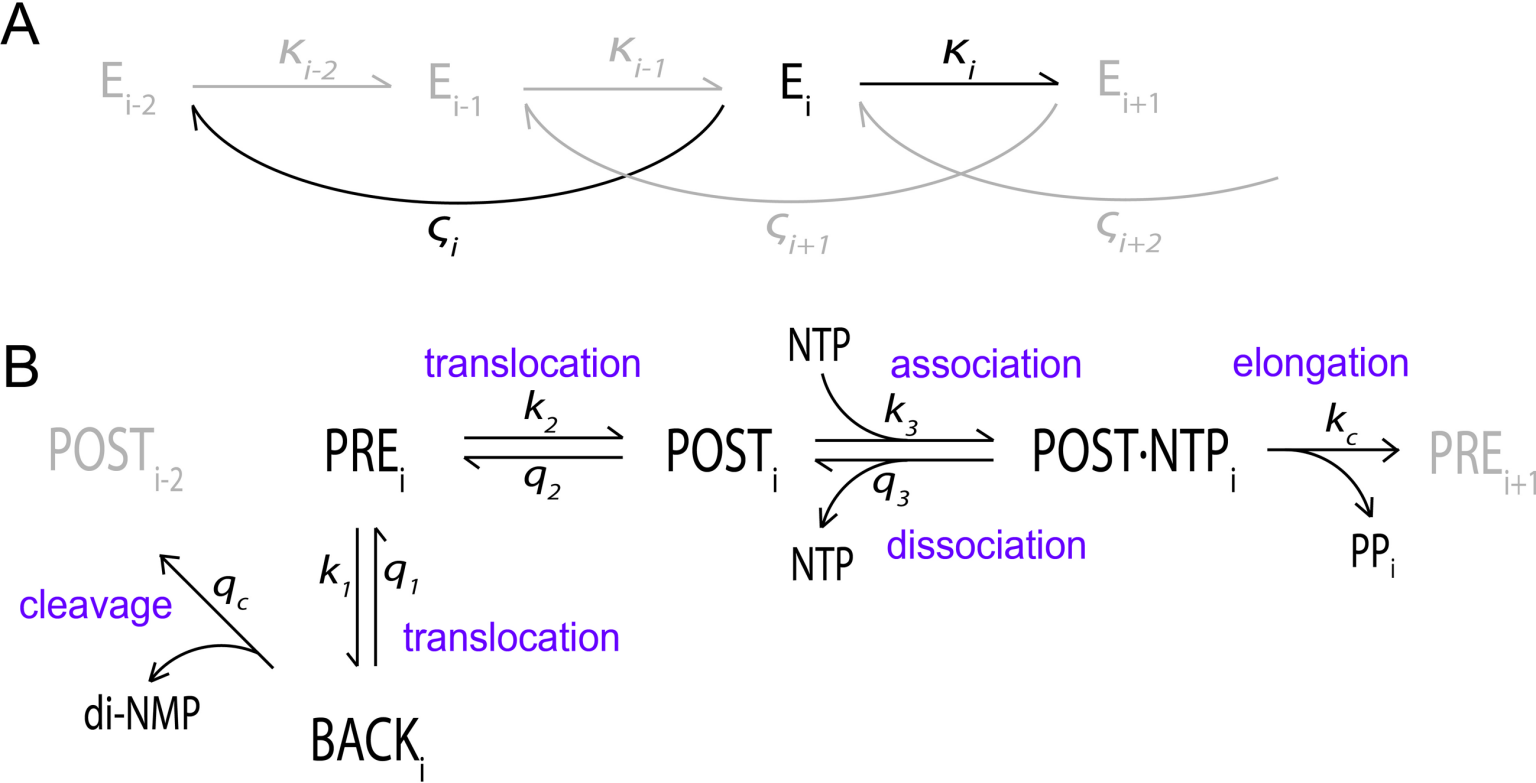
The transcription model. (**A**) The elongation states, defined by the length of the transcript. The transcript grows and dinucleotides are cleaved off with compound rate constants *κ*_i_ and *ς*_i_, respectively, which are functions of the rate constants of the sub-states of the transcription cycle. (**B**) The sub-states of the nucleotide addition cycle with backstepping and transcript cleavage. The rate constants of the reactions connecting the sub-states are indicated.

Our approach is based on preceding mathematical models that have outlined the essential reactions of transcript elongation; translocation, nucleotide association and dissociation, phosphodiester bond formation and transcript cleavage. The standard model of transcription, used by Bai *et al.* (14), Guajardo and Sousa (15) and others, is sketched out in Figure 1. The length of the growing transcript defines the current elongation state, representing 1 nt addition cycle (16). We have previously extended the standard model to include also transcriptional accuracy with two nucleotide selection steps, one initial selection and one proofreading selection step, which proved to be in line with experimental evidence (10).

Inspection of this model in conjunction with previous experimental results has led to the realization that the polymerase can utilize the same proofreading mechanism for two consecutive proofreading selection steps. This follows naturally from established experimental results: (i) during transcript cleavage the RNA polymerase always cleaves off an RNA fragment of at least 2 nt (17); (ii) general properties of base stacking in double-stranded nucleic acids suggest that a mismatched base pair de-stabilizes the base-pairing interactions of both its nearest neighbors (13); (iii) incorporation of a correct nucleotide is slowed down if the previously incorporated nucleotide is incorrect (18,19). Together, these assertions suggest that the polymerase can detect the instability of the base pair following a misincorporation, and discard the erroneous base by dinucleotide cleavage.

In the present work we describe the two-step transcriptional proofreading that follows from the standard model of transcription. We also present an improved model for initial selection and a first proofreading step that is followed by a second proofreading step. In addition, we describe how transcript cleavage allows for further rounds of proofreading due to polymerase revisiting of previously visited positions and estimate the resulting error reduction.

## MATERIALS AND METHODS

### Enzymatic accuracy theory

The total accuracy of a system of enzymatic reactions is here defined as the ratio of the flows of product formation, using correct (*j*^c^) or incorrect (*j*^nc^) substrates. The flow of product formation can be expressed in terms of efficiency of product formation (k_cat_/K_m_) and enzyme and substrate concentrations ([S^c/nc^]).

(1) \begin{equation*}{A_{{\rm tot}}}([S]) = \frac{{{j^{\rm c}}}}{{{j^{{\rm nc}{\rm }}}}} = \frac{{E[{S^{\rm c}}]{{\left( {\frac{{{k_{{\rm cat}}}}}{{{K_{\rm m}}}}} \right)}^{\rm c}}}}{{E[{S^{{\rm nc}}}]{{\left( {\frac{{{k_{{\rm cat}}}}}{{{K_{\rm m}}}}} \right)}^{{\rm nc}}}}}\end{equation*}

Here and below, the superscripts c and nc are notations for the substrate being cognate or non-cognate to the template. The normalized accuracy *A*, equal to *A*_tot_ in Equation (1) when [*S*^c^] = [*S*^nc^], is the ratio of efficiency (*k*_cat_/*K*_m_) of product formation from a correct substrate and a particular incorrect substrate. This can equivalently be expressed as the ratio of products of association rate constants, *k*_a_, and probabilities *P* of product formation from correct and incorrect substrates after their first encounter with the enzyme:

(2) \begin{equation*}A = \frac{{{{\left( {{{{k_{{\rm cat}}}} \mathord{\left/ {\vphantom {{{k_{{\rm cat}}}} {{K_{\rm m}}}}} \right. } {{K_{\rm m}}}}} \right)}^{\rm c}}}}{{{{\left( {{{{k_{{\rm cat}}}} \mathord{\left/ {\vphantom {{{k_{cat}}} {{K_{\rm m}}}}} \right. } {{K_{\rm m}}}}} \right)}^{{\rm nc}}}}} = \frac{{k_{\rm a}^{\rm c}{P^{\rm c}}}}{{k_{\rm a}^{{\rm nc}}{P^{{\rm nc}}}}}\end{equation*}

With the two options for the enzyme substrate complex of product formation at rate constant *k* and substrate dissociation at rate constant *q*, the normalized accuracy *A* can be expressed in terms of the reaction rate constant ratios *q/k* for cognate and non-cognate substrates, when the association rate constant is the same for both substrates.

(3) \begin{equation*}A = \frac{{{k_{\rm a}{\rm }}{P^{\rm c}{\rm }}}}{{{k_{\rm a}}{P^{{\rm nc}}}}} = \frac{{{P^{\rm c}}}}{{{P^{{\rm nc}{\rm }}}}} = \frac{{{{\left( {\frac{k}{{k + q}}} \right)}^{\rm c}}}}{{{{\left( {\frac{k}{{k + q}}} \right)}^{{\rm nc}}}}} = \frac{{1 + {{\left( {\frac{q}{k}} \right)}^{{\rm nc}}}}}{{1 + {{\left( {\frac{q}{k}} \right)}^{\rm c}}}}\end{equation*}

Discrimination against non-cognate substrates thus occurs when the ratios *q/k* of reaction rate constants are different for cognate and non-cognate substrates. The maximum discrimination *d* between two substrates is the maximum accuracy of the reaction. Linus Pauling calculated the maximum discrimination *d* between two substrates cognate and non-cognate to the template based on the interaction energy as:

(4) \begin{equation*}d = {e^{ - (\Delta {G^d})/(R \cdot T)}}\end{equation*}

(20,21), where *ΔG* is the difference in free energy of the interaction between the template and correct and incorrect substrates. In transcription, the maximum discrimination was estimated by Pauling’s equation to 10^2^–10^3^, using a *ΔG^d^* value of −2.7 to −4.4 kcal/mol (22). There is, however, a problem with this accuracy calculation. According to Michaelis–Menten kinetics, the system can achieve the maximum discrimination *d* only when free and complex bound substrates are in equilibrium; i.e. when the cognate reaction rate approaches zero (7,23), which is never the case due to the selection pressure for reaction speed on the enzyme. Instead, the polymerase operates somewhere between the minimum accuracy value 1 and the maximum discrimination *d* (24). (For a more detailed explanation on accuracy theory, see (25).)

### The standard model of transcript elongation

The basic reactions of transcript elongation are outlined above and in Figure 1. Transcript elongation is commonly modeled as a series of stochastic translocations, where the polymerase jumps forward and backward along the template DNA with a net movement that is driven in the forward direction by thermodynamically favorable nucleotide addition in the forward-translocated state (10,14,15). There are also different types of backtracking events in transcript elongation, related to proofreading and transcription elongation pausing. While multiple-step backward translocation, or ‘long’ backtracking, seems to be a type of transcriptional arrest that occurs only from specific positions along the template (26,27), one-step backward translocation or *backstepping*, is a fast and common event from apparently any position (18,28). Structural data indicate that the backstepped state is stabilized by the insertion of the protruding last incorporated nucleotide in a binding pocket (29). From these data we conclude that backstepping can promote proofreading at a large set of positions along the template and therefore is essential for the proofreading mechanism, while long backtracking from a small number of specific positions is not. The notion that backstepping and long backtracking may have distinct roles in transcription elongation is in line with the observation that different cleavage factors assist in the two cases; bacterial cleavage factor GreA cleaves off di- and tri-nucleotides, and GreB cleaves off longer RNA segments (30).

Transcript cleavage always releases a sequence of 2 nt or longer, corresponding to the number of preceding backward translocations plus one (Figure 1). This was observed already with the discovery of the intrinsic cleavage function in bacterial RNA polymerase (17) and has since then been observed in both eukaryotes (31) and archaea (32). This feature of transcript cleavage was only recently included in transcript elongation modeling (10) and thus its effect might have been overlooked. In our model where only backstepping is allowed, the cleavage product is always a dinucleotide.

Each elongation state in the chain of elongation reactions, denoted E_i_ in Figure 1A and defined by its transcript length *i*, comprises four sub-states of the polymerase: the pre-translocated state, PRE; the forward-translocated state, POST; the forward-translocated state with a nucleotide nucleoside triphosphate (NTP) in the active site, POST·NTP, where phosphodiester bond formation may occur; and the backstepped state, BACK, in which transcript cleavage may take place. The reactions between the sub-states of the transcription cycle are modeled as first or second order rate constants, where the latter are multiplied by external nucleotide concentrations for the nucleotide association steps. For nucleotide association, therefore, the reaction rate is a reaction rate constant multiplied by the nucleotide concentration, which results in a pseudo first order rate constant. We use *κ* and *ς* for the compound rate constants of reactions between elongation states, defined from the first or pseudo first order rate constants of the sub-states.

As in previous transcription models (14,33), simple rate constants for state-connecting reactions are calculated from the Eyring equation with a generic reaction rate constant *k_1→2_* of going from state 1 to state 2 given by:

(5) \begin{equation*}{k_{1 \to 2}} = {k_{{\rm pre}}} \cdot {e^{ - (\Delta {G^\ddagger } + \max ((\Delta {G_2} - \Delta {G_1}),0))/(R \cdot T)}}\end{equation*}

where, *k*_pre_ is a pre-factor constant, *R* is the gas constant, *T* is the absolute temperature and the *ΔG* terms account for the highest free energy on the passage from state one to state two, i.e. the transition state. The term *ΔG^‡^* is a fixed free energy barrier for each type of reaction and is listed in Supplementary Table S1 for all reactions. The terms *ΔG_1_* and *ΔG_2_* are the standard free energies of ground states one and two, respectively. If the reaction is going from a ground state to another with a higher standard free energy, the free energy difference between the two states must also be included in the transition state. The difference in total free energy of the two sub-states, (*ΔG_2_ – ΔG_1_*), is hence included in the equation if *ΔG_2_* > *ΔG_1_* but omitted if *ΔG_2_* < *ΔG_1_*. Since the energy barriers *ΔG^‡^* are fixed and common for all template sequences, the free energy differences between the sub-states are the only source of the template-dependent accuracy variation in the model. The total free energy of each sub-state in the transcription model is defined as the sum of the free energies of the nucleic acids in the transcription bubble and the polymerase (33):

(6) \begin{equation*}\Delta {G_{{\rm state}}} = \Delta {G_{{\rm DNA}/{\rm DNA}}} + \Delta {G_{{\rm RNA}/{\rm DNA}}} + \Delta {G_{{\rm pol}}}\end{equation*}

Here, *ΔG*_DNA/DNA_ is the free energy cost of opening the double stranded DNA to form the transcription bubble, *ΔG*_RNA/DNA_ is the free energy gained upon formation of the RNA/DNA hybrid and *ΔG*_pol_ is the free energy contribution from the polymerase in its interactions with the transcription bubble. The notation for Gibbs free energy here includes ‘Δ’ for the ground states, to signify that this free energy of the state is not the total free energy of the complex but a difference in free energy relative to some level of free energy common to all ground states. Free energy barriers *ΔG^‡^* are added to the ground states for the free energy of activation that is needed for the reactions to occur.

The term *ΔG*_pol_ includes stabilization of the transcription bubble, catalysis of reactions, and discrimination between correct and non-correct substrates. The polymerase stabilization of the transcription bubble is assumed to be state and sequence independent and thus to cancel out in the difference between *ΔG*_state_ of two sub-states, which determines the reaction rate constant. The other effects of the polymerase, reaction catalysis and mismatch discrimination, are included in the reaction barriers and other parameters presented in Supplementary Table S1. The free energy *ΔG*_state_ of each sub-state is hence completely specified by the particular DNA and RNA sequences in its transcription bubble.

The transcription bubble consists of 12 base pairs of denatured double-stranded DNA and 8–9 bp of RNA/DNA hybrid (16,34). The free energy of the transcription bubble is described by the sum of the free energy required to break or form the hydrogen bonds between opposing bases of the nucleic acid sequences (33), using experimentally determined nearest-neighbor parameters for double-stranded DNA (11) and RNA/DNA hybrid (12). Nearest-neighbor parameters can accurately predict nucleic acid melting energies (11) by including the effect of base stacking of adjacent base pairs on the stability of the hydrogen bonds between the bases in the 2 bp. This is why the nearest-neighbor parameters are defined for pairs of base pairs, and the free energy of a sequence is calculated by summation of the free energies of its pairs of base pairs. Yet again, since only the difference in *ΔG*_state_ between two states is used in the rate constant calculation, only pairs of base-pairs that differ between two sub-states will affect the reaction rate constants that connect the two states (further discussed below).

The reaction rate barriers (Supplementary Table S1) are compatible with available experimental data and have been tuned to match the *in vivo* estimated time of 60 s to transcribe the ribosomal RNA operon *rrnC* (35). Supplementary Figure S1 and Table S2 compare a few different parameter sets.

### Accuracy calculations in transcription

**Figure 2.**
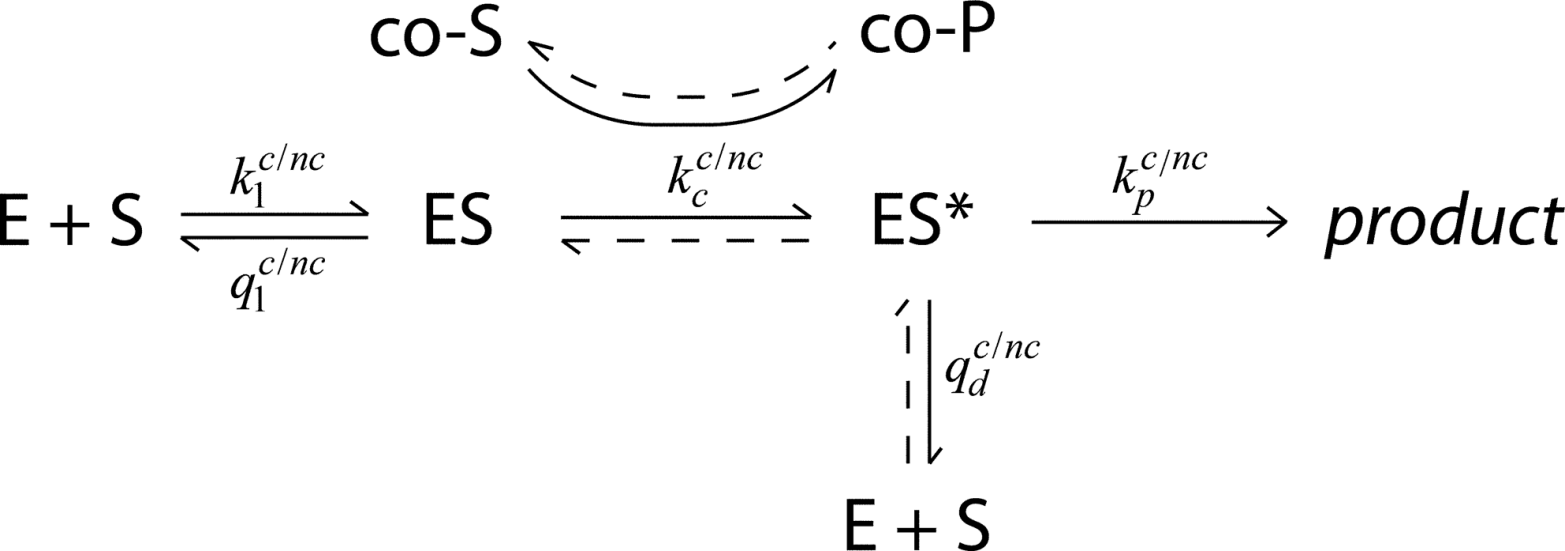
A general scheme of kinetic proofreading. A substrate S is turned into product by an enzyme E. The substrate associates to the enzyme with an association rate constant *k*_1_, after which the complex could either dissociate with a dissociation rate constant *q*_1_ or form a high energy intermediate complex ES*. The formation of ES* is facilitated by the enzymatically coupled processing of the co-substrate co-S to form a co-substrate co-P. The high energy intermediate ES* may either dissociate with a rate constant *q*_d_ or continue to product formation with a rate constant *k*_p_. The formation of the intermediate ES* is energetically driven by the shift in concentration of co-S far above equilibrium with co-P.

The comparison of substrate interactions in Pauling’s calculation corresponds to the initial selection of substrates after nucleotide association, but, unknown in the earliest studies on RNA polymerase accuracy, there is also a kinetic proofreading mechanism for transcription. Kinetic proofreading, first described by Hopfield (36) and Ninio (23), requires a second, thermodynamically driven substrate exit reaction (vertical reaction step in Figure 2). Due to the detailed balance constraint a thermodynamic driving force is necessary to prevent an otherwise obligatory substrate influx along the intended exit path (37,38). These principles are illustrated by the generic scheme in Figure 2, where the initial and second substrate exit reactions have rate constants *q*_1_ and *q*_d_, respectively. All reactions could potentially have discriminating reaction rate constants for cognate and non-cognate substrates, why all rate constants are marked c/nc in Figure 2, but only one discriminating reaction is required for initial and proofreading selection, respectively.

The existence of proofreading in transcription is supported by the experimental observations that (i) mismatch insertions increase polymerase backtracking; (ii) transcript cleavage introduces mismatch discrimination; (iii) transcript cleavage rescues backtracked complexes for continued elongation (18,39–41). This would suggest that the second substrate exit path in a putative proofreading mechanism is the endolytic transcript cleavage by the RNA polymerase after polymerase backtracking (17), whereupon the cleavage product is further degraded to nucleoside monophosphates and pyrophosphate. The proofreading mechanism requires that the concentration of degraded cleavage reaction products is shifted far below equilibrium with substrate concentration. Ultimately, it would be the shift in equilibrium of nucleoside triphosphates over nucleoside monophosphates and pyrophosphate that confers the driving force for proofreading in transcription (25). Although the existence of proofreading in transcription is fairly well established, quantitative data on how much kinetic proofreading contributes to transcriptional accuracy are still missing.

The early calculations of the maximum substrate discrimination in transcription in Equation (4) had another flaw, besides that enzymes due to kinetic loss cannot operate at the maximal accuracy near equilibrium with their substrates, in that the estimated *ΔG*^d^ value of a transcription mismatch was later replaced by more accurate experimental measurements of a much smaller difference between correct and mismatched base pairs (12,42). Strangely enough, the old inflated *ΔG*^d^ estimate is still in use in transcriptional accuracy modeling (43–45). Using recent numbers, the average energy difference between a correct and a last position mismatched RNA/DNA hybrid is −0.9 kcal/mol (12,13), rather than −2.7 to −4.4 kcal/mol, which makes the maximum discrimination only 4.3.

Fortunately, transcriptional fidelity of substrate selection is saved by stereospecific discrimination against mismatches. The free energy differences between correct and mismatched complexes above were measured in solution (11–13), but the free energy difference is amplified in complex with the polymerase. This accuracy amplification is conferred partly by folding of the trigger loop, a flexible domain in the active centre of RNAP (46). Its folding closes the active centre and increases the accuracy of nucleotide selection by an induced-fit mechanism against non-complementary nucleotides (47,48). The trigger loop is also an integral part of the polymerase dependent mechanism for transcript cleavage (41), but can in the active centre be replaced by the associated cleavage factors GreA and GreB (49).

In our model, the increase in selection bestowed by the polymerase is implemented through the mismatch discriminating *polymerase effect* of a factor 50. The polymerase effect is applied only if the last or penultimate incorporation is mismatched, favoring cognate in relation to non-cognate substrates in phosphodiester bond formation (*k*_c_) and disfavouring cognate transcript cleavage (*q*_c_). A uniform factor 50 is used due to lack of data on the putative variability of the suggested accuracy enhancing mechanisms.

In addition, the stabilities of mismatched complexes in the state BACK have been increased as in a previous transcription model (45), if the mismatch is in the last or penultimate position. This is motivated by the notion that the backtrack binding pocket is more accessible for misincorporations (29).

### Two-step proofreading

The effect of adjacent bases, as it has been understood so far, is that base stacking interactions of the aromatic rings of DNA and RNA significantly stabilize or de-stabilize the hydrogen bonds to the opposite bases. In transcript elongation an incoming nucleotide and the template nucleotide in the active site form a base pair that interacts with the preceding base pair. The difference in standard free energy between nucleotide substrates in base pair formation is therefore determined by their interactions with the template nucleotide, tuned by the base stacking to the preceding base pair. Using nearest-neighbor parameters to predict the melting energy of a double-stranded sequence, base stacking is taken into account by summarizing pairs of base pairs. Each base pair is thus part of two pairs of base pairs, if not at the end of the string.

A mismatched base pair directly affects and is affected by its two neighboring base pairs as well. The first neighbour, that the incoming base interacts with before transcript elongation, directly affects the initial selection by the stacking effect on the interaction energy of the mismatched base pair. This standard free energy difference, the melting energy of the pair of base pairs formed by the two last incorporated nucleotides, is evaluated and in case of a mismatch discriminated against in initial selection. It determines the maximal accuracy of initial selection, the *d*-value, by which a cognate base is favoured in relation to non-cognate competitors (25). Thus, the mismatched base pair and its neighbouring base pair are the only base pairs that affect the initial selection.

The proofreading selection is governed by the same pair of base pairs as initial selection, i.e. the base pair with a misincorporation and its preceding neighbor, but also by all other base pairs in the transcription bubble that affect the probability of transcript cleavage (Equation 8). Among these is the base pair after a misincorporation that is formed more slowly than after a correct incorporation (18,19), in agreement with the prediction by the nearest-neighbor model. The slowing of incorporation after an error is a consequence of the instability of the state POST*NTP (post-translocated with an associated nucleotide) compared to the state without the associated nucleotide (POST). The state POST is generally stabilized by the formation of the new base pair consisting of the template DNA and the incoming nucleotide. When the previous incorporation is a mismatched base pair, this stabilizing energy of the next base pair is reduced due to base stacking. The state POST*NTP with a mismatch in the penultimate position hence becomes less stable, and the probability for nucleotide dissociation higher, than for the cognate case.

**Figure 3.**
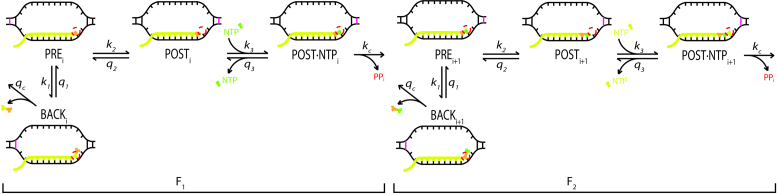
Two-step proofreading with transcription bubbles. The states and reactions are the same as in Figure 1. F_1_ corresponds to the first proofreading step and F_2_ to the second. In F_1_, a misincorporated base (orange) is in the last position of the transcript, and can be cleaved off or extended by next base incorporation (green). In F_2_, the previously misincorporated base (orange) can again be cleaved off, or extended by a next base incorporation (yellow), that is unaffected by the presence of the misincorporated base. The DNA is shown in black with 2 bp in magenta that mark the bounds of the involved base pairs in the two steps of proofreading. The RNA is shown in yellow, with the nucleotide subject to proofreading in orange and the next incorporated nucleotide in green.

But what about the following elongation cycle? We first note that in a transcription elongation cycle following the proofreading of a misincorporation, the interaction free energy of the last incorporated base pair is expected to be higher (meaning the interaction is less stable) than if the preceding base pair had been correct, thus increasing the propensity to backtrack. In more detail, the mismatch in a previous cycle destabilizes the PRE state compared to the BACK state since the RNA/DNA hybrid in state PRE contains two pairs of base pairs with a mismatch and that of BACK only one (Figure 3). Furthermore, nucleotide cleavage by the RNA polymerase always removes *two* base pairs after only one step of backtracking, meaning that the polymerase can remove a mismatch by detection of its destabilizing effect on either one of its two neighbours. In the backstepped state, the flawed and the following base pair will both be cleaved off (Figure 3).

From these considerations we propose that the accuracy of template dependent nucleotide selection by RNA polymerase is maintained by one initial selection step and two proofreading steps (Figure 3). In the first proofreading step, a misincorporation at the transcript position *n* can be corrected in elongation state *E*_n_ due to its enhanced backtracking propensity and reduced rate of entry into the next cycle. If the misincorporation escapes the first proofreading step, it can be removed in the second proofreading step in elongation state *E*_n+1_ due to its enhanced backtracking propensity. The second proofreading step is identical to the first but for one difference: In the first proofreading step, the elongation is impaired so that the selectivity originates both from a decreased forward rate and an increased backward rate for mismatches. In the second proofreading step, however, the selectivity relies only on the increased backward rate as the forward reaction is a correct incorporation next to another correct incorporation. The second step of proofreading means that using the same cleavage mechanism, the polymerase gains an additional proofreading check, without any need to determine whether the misincorporation is in the first or second position of the dinucleotide cleavage product. Each elongation state *E*_i_ along a transcribed sequence hence constitutes two proofreading events, examining nucleotides i and i-1 at the same time. Both proofreading steps of nucleotide incorporation are outlined in Figure 3.

### Transcriptional accuracy in the model

The accuracy of initial nucleotide selection, *I*, is the probability that a cognate RNA polymerase–nucleotide complex undergoes phosphodiester bond formation divided by that of a non-cognate complex. These probabilities for elongation state *E*_i_, expressed in terms of the ratio (*q/k*)_i_ of backward-to-forward reaction rate constants (Equation 3), are *q_3_/k_c_* (Figure 1B), the nucleotide dissociation rate constant *q*_3_ divided by the rate constant *k*_c_ for phosphodiester bond formation (25):

(7) \begin{equation*}I = \frac{{{k_{\rm a}}P_I^{\rm c}}}{{{k_a}P_I^{{\rm nc}}}} = \frac{{P_I^{\rm c}}}{{P_I^{{\rm nc}}}} = \frac{{{{\left( {\frac{k}{{k + q}}} \right)}^{\rm c}}}}{{{{\left( {\frac{k}{{k + q}}} \right)}^{{\rm nc}}}}} = \frac{{1 + {{\left( {\frac{q}{k}} \right)}^{{\rm nc}}}}}{{1 + {{\left( {\frac{q}{k}} \right)}^{\rm c}}}} = \frac{{1 + {{\left( {\frac{{{q_{\rm 3}}}}{{{k_{\rm c}}}}} \right)}^{{\rm nc}}}}}{{1 + {{\left( {\frac{{{q_{\rm 3}}}}{{{k_{\rm c}}}}} \right)}^{\rm c}}}}{\rm{ }}\end{equation*}

The proofreading selection spans the entire next elongation step. The accuracy enhancement for a position i by the first step of proofreading, *F*_1_, is defined as the ratio of the probabilities of product formation of cognate and non-cognate substrates. The probability of product formation in proofreading is the probability that a nucleotide in the last position i of the transcript escapes transcript cleavage in elongation state *E*_i_ and instead remains for the next elongation. This probability, again formulated as a ratio of the backward and forward reaction rate constants, is the ratio (*ς/κ*)_i_ of the cleavage and elongation compound rate constants of *E*_i_ (Figure 1A), composed of all the sub-state reaction rate constants within the elongation state (25).

(8) \begin{equation*}\begin{array}{@{}*{1}{l}@{}} {{F_{1{\rm i}}} = {{\left( {\frac{{P_F^{\rm c}{\rm }}}{{P_F^{{\rm nc}}}}} \right)}_i} = \frac{{\left( {\frac{\kappa }{{\kappa + \varsigma }}} \right)_{\rm i}^{\rm c}}}{{\left( {\frac{\kappa }{{\kappa + \varsigma }}} \right)_i^{{\rm nc}}}} = \frac{{1 + \left( {\frac{\varsigma }{\kappa }} \right)_i^{{\rm nc}}}}{{1 + \left( {\frac{\varsigma }{\kappa }} \right)_i^{\rm c}}};}\\ {{\rm{where}}\;\left( {\frac{\varsigma }{\kappa }} \right)_i^{{\rm c}/{\rm nc}} = {{\left( {\frac{{k_1^{{\rm c}/{\rm nc}}}}{{{k_2}}}\frac{{1 + \frac{{{q_2}}}{{{k_{\rm 3}{\rm }} \cdot [{\rm NT}{\rm }{{\rm P}_{{\rm i} + 1}}]}}\left( {1 + \frac{{q_{\rm 3}^{{\rm c}/{\rm nc}}}}{{k_{\rm c}^{{\rm c}{\rm }/{\rm nc}}}}} \right)}}{{1 + \frac{{{q_1}^{c/nc}}}{{q_{\rm c}^{{\rm c}/{\rm nc}{\rm }}}}}}} \right)}_i}} \end{array}\end{equation*}

Like before, the superscript c/nc marks the reaction rate constants that are affected by a misincorporation. The reaction rate constant calculations (Equation 5) compare the transcription bubble energies of the initial and final states of the reaction, as described here and previously (‘Materials and Methods’ section, (10)). When the cognate free energy difference between two reaction states is different from that of the non-cognate difference, the cognate and non-cognate rate constants are also different (Supplementary Table S1), which is how the polymerase can recognize an error from the interaction energy with the template. The affected reaction rate constants are hence the discriminating reactions; translocation between PRE and BACK (*k*_1_ and *q*_1_) and nucleotide dissociation (*q*_3_). The reaction rate constants of transcript cleavage (*q*_c_) and phosphodiester bond formation (*k*_c_) are not affected by differences in the transcription bubble, but defined only by their mismatch discriminating rate barriers, described below and in Supplementary Table S1.

To calculate the reaction rate constants of the non-cognate substrates, mismatch nearest-neighbour parameters are used for the base pairs in the position of the mismatch instead of correct nearest-neighbour parameters. The published set of misincorporation nearest-neighbour parameters only includes four; A·A, C·C, G·G and U·T; out of all twelve possible mismatches, yet with all possible 3′ and 5′ neighbors (13). We have therefore approximated the rest of the mismatches with the only available mismatch, until a full dataset is available.

The accuracy enhancement for a position i by the second proofreading selection, *F*_2_, is the probability that an incorporated cognate base in the last position i of the transcript escapes transcript cleavage in elongation state *E*_i+1_ divided by that of a non-cognate base. These probabilities are expressed in terms of the ratio (*ς/κ*)_i+1_ of the compound cleavage and elongation rate constants of the state *E*_i+1_, determined by the sub-state rate constants in *E*_i+1_ (Figure 1A):

(9) \begin{equation*}\begin{array}{@{}*{1}{l}@{}} {{F_{2i}} = \frac{{1 + \left( {\frac{\varsigma }{\kappa }} \right)_{{\rm i} + 1}^{{\rm nc}}}}{{1 + \left( {\frac{\varsigma }{\kappa }} \right)_{{\rm i} + 1}^{\rm c}}};}\\ {{\rm{where}}\;\;\left( {\frac{\varsigma }{\kappa }} \right)_{{\rm i} + 1}^{{\rm c}{\rm }/{\rm nc}} = {{\left( {\frac{{k_1^{{\rm c}{\rm }/{\rm nc}}}}{{{k_2}}}\frac{{1 + \frac{{{q_2}}}{{{k_{\rm 3}}[{\rm NT}{{\rm P}_{{\rm i }+ 2}}]}}\left( {1 + \frac{{{q_{\rm 3}}}}{{{k_{\rm c}}}}} \right)}}{{1 + \frac{{{q_1}^{{\rm c}/{\rm nc}}}}{{q_c^{{\rm c}/{\rm nc}}}}}}} \right)}_{{\rm i} + 1}}} \end{array}\end{equation*}

We note that for *F*_1i_, parameters *q*_3_ and *k*_c_ in *E*_i_ are sensitive to whether the incorporated base i is cognate or non-cognate. For *F*_2i_, by contrast, parameters *q*_3_ and *k*_c_ in *E*_i+1_ are insensitive to whether the incorporated base i is cognate or non-cognate, since the base pair formed by the incoming nucleotide and the template base have a correct base pair as the nearest neighbor (as the model assumes that there cannot be two consecutive mismatches). The accuracy amplification is therefore expected to be larger in the first than in the second proofreading step.

The normalized accuracy *A* of an RNA polymerase with initial selection *I*, proofreading selection *F*_1_ and proofreading selection *F*_2_ at a given template position can be written$A = ( {P_I^{\rm c}/P_I^{{\rm nc}}} ) \cdot ( {P_{F1}^{\rm c}/P_{F1}^{{\rm nc}}} ) \cdot ( {P_{F2}^{\rm c}/P_{F2}^{{\rm nc}}} ) = I \cdot {F_1} \cdot {F_2}$. The total accuracy *A*_tot_ of the polymerase incorporating one cognate over three non-cognate nucleotides at a given template position, defined as the cognate incorporation probability divided by the sum of non-cognate incorporation probabilities, is hence:

(10) \begin{equation*}\begin{array}{@{}*{1}{l}@{}} {{A_{{\rm tot}}}\left( {[{S^{\rm c}}],[{S^{{\rm n}{{\rm c}_1}}}],[{S^{{\rm n}{{\rm c}_2}}}],[{S^{{\rm n}{{\rm c}_3}}}]} \right)}\\ { = \frac{{[{S^{\rm c}}]P_I^{\rm c}P_{F1}^{\rm c}P_{F2}^{\rm c}}}{{[{S^{{\rm n}{{\rm c}_1}}}]P_I^{{\rm n}{{\rm c}_1}}P_{F1}^{{\rm n}{{\rm c}_1}}P_{F2}^{{\rm n}{{\rm c}_1}} + [{S^{{\rm n}{{\rm c}_2}}}]P_I^{{\rm n}{{\rm c}_2}}P_{F1}^{{\rm n}{{\rm c}_2}}P_{F2}^{{\rm n}{{\rm c}_2}} + [{S^{{\rm n}{{\rm c}_3}}}]P_I^{{\rm n}{{\rm c}_3}}P_{F1}^{{\rm n}{{\rm c}_3}}P_{F2}^{{\rm n}{{\rm c}_3}}}}} \end{array}\end{equation*}

If we denote the total accuracy at template position i as *A*_toti_, the error frequency at position i, Err_i_, is given by:

(11) \begin{equation*}{\rm{Er}}{{\rm{r}}_{\rm i}} = \frac{1}{{1 + {A_{{\rm toti}}}}}\end{equation*}

In the results below, a polymerase effect has been added to the model by letting the polymerase discriminate between correct and incorrect base pairing in the transition state of the phosphodiester bond formation and in the transition state of the transcript cleavage. As described above, this polymerase effect accounts for any accuracy enhancing property of the polymerase, such as hindrance of phosphodiester bond formation for mismatched base pairs and of transcript cleavage for correct base pairs. We note that the polymerase effect in the two reactions may be substrate specific, but in lack of data we use the discriminating factor 50 for all base pairs. However, since it alters the reaction rate constants *k*_c_ and *q*_c_ that are tuned by the other reaction rates in the accuracy calculations (see Equations 8 and 9, and Equations 10 and 11 in the Supplementary Data), the ultimate effect of the polymerase discrimination depends on the template sequence. Without the mismatch discrimination by the polymerase, most of the proofreading accuracy variation is truncated at 1, the minimum discrimination.

In the first proofreading step, the polymerase effect acts on both the rate constant of phosphodiester bond formation (*k*_c_) and the rate constant of transcript cleavage (*q*_c_) to increase the accuracy. In the second proofreading step, *k*_c_ is not affected by the mismatch discrimination of the polymerase since the scrutinized pair of base pairs, formed by the last incorporated base pair and incoming nucleotide and its template, is correct. The only accuracy amplification is on the transcript cleavage and the stabilization of the backstepped state for misincorporations, why the proofreading selection of proofreading step two is expected to be lower than that of the first step.

### Revisiting positions

In the above description of the accuracy there is a simplification in the expression of the processing probability of the substrate in Equation (3). We assumed that there are only two options—the forward and the backward reaction, signifying product formation and substrate rejection. The definitive product formation, however, is not attained until the whole operon is transcribed. We cannot know in advance whether an incorporated nucleotide will appear in the final transcript, or if it will later be cleaved off through repeated backtracking and cleavage by the polymerase.

Every time a position is revisited due to transcript cleavage from a downstream position, the last nucleotides of the transcript undergo additional rounds of proofreading selection; a round of first-step proofreading for the last nucleotide and a round of second-step proofreading for the penultimate nucleotide. Therefore, we have calculated for every position the occurrence of revisits depending on the propensity of cleavage in the downstream positions, and how these extra rounds of proofreading enhance the total accuracy per position. The details of the calculations are found in the Supplementary Data.

### Total transcription time

The total transcription time was in our previous publication (10) calculated by solving the equation system of the integrated master equation, and this method is used also in this paper. However, the old equation system only contains the cognate reaction rate constants, and it was assumed as a simplifying approximation that the polymerase always arrives at an elongation state in the pre-translocated sub-state, also after transcript cleavage. With the new master equation including the double elongation states presented in Supplementary Figure S2 and the non-cognate reaction rate constants, the transcription time calculated here is a better representation of the *in vivo* transcription time. The calculations are described in detail in the Supplementary Data.

## RESULTS

The suggestion of the two-step proofreading is based on experimental observations and the standard model of transcription. In order to calculate the effect on the overall error frequency of the second step of proofreading, we extended our previous model of transcriptional accuracy with a second proofreading step and refined the model to give a better estimate of the total transcription time.

We calculated the accuracy for initial selection and two steps of proofreading for an example sequence, the ribosomal operon C from *E. coli, rrnC*, of 5552 bp (50). The transcription of the *rRNA* operons has been studied experimentally (35,51), and the transcripts lacks trailing ribosomes that could interrupt backtracking. All calculations were performed in MATLAB 7.9.0 (The MathWorks, MA, USA).

**Figure 4.**
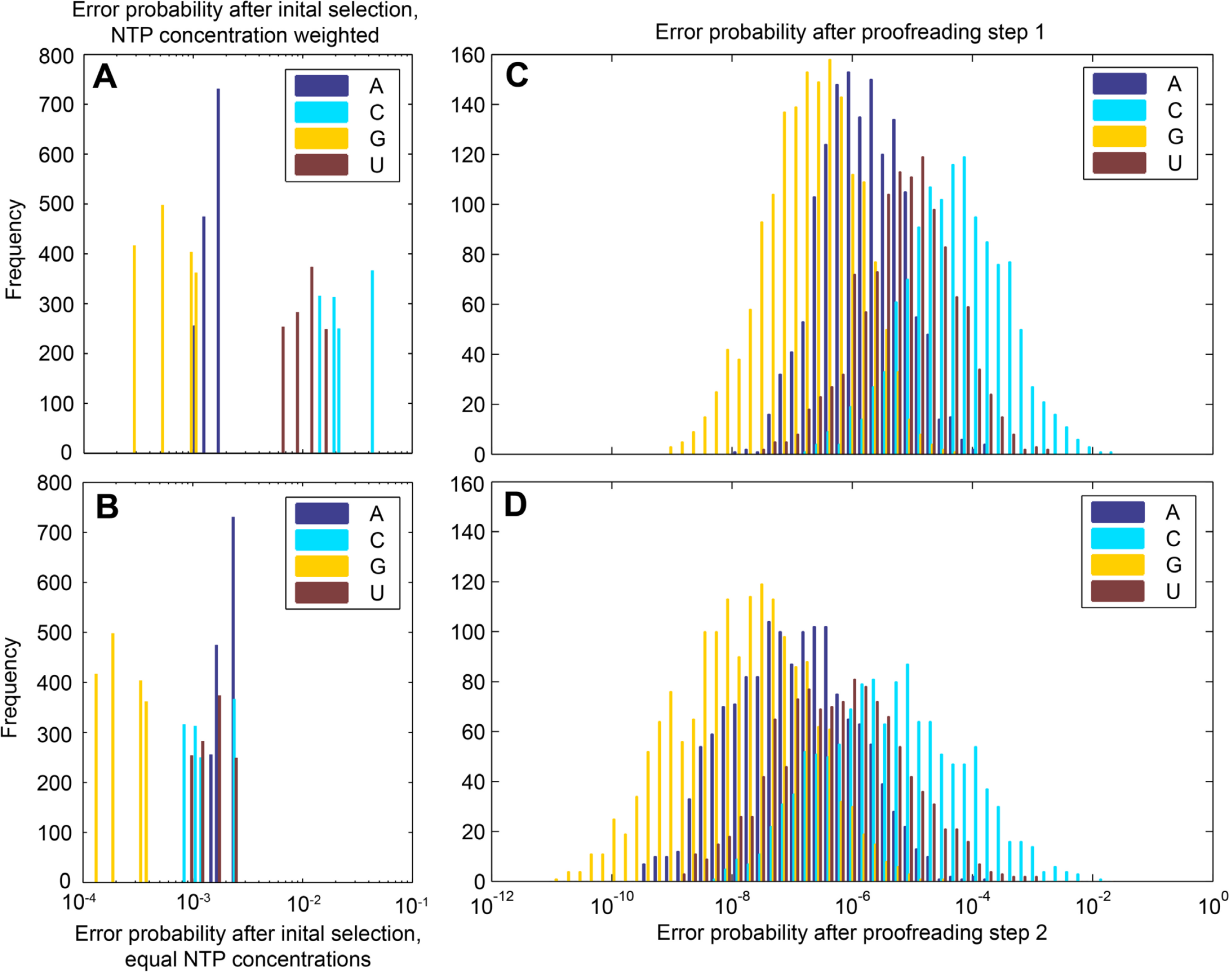
Histograms of error probability after initial selection, proofreading step 1 and proofreading step 2 in *rrnC*. The histograms show the frequency of positions within the operon with en error probability within the scope of the bar. The data are grouped by the cognate substrate X, and with only one type of mismatch per template (A·A, C·C, G·G or U·T), panels **A**–**C** represents the error probability of the mismatch Y·Y compared to the correct X·Y. (A) Error probability after initial selection. The distribution is discrete, since the variation comes from only 2 bp. The error probability is nucleotide concentration weighted to show the probabilities of errors before proofreading. (B) Error probability after initial selection without nucleotide concentration weighting, representing the initial selection discrimination as expressed in Equation (7). (C) Error probability after initial selection and the first step of proofreading. This proofreading step gets the benefit of both polymerase effects. (**D**) Error probability after initial selection and two steps of proofreading. The second proofreading step is enhanced only by the polymerase effect on cleavage. The accuracy amplification by revisiting positions is not included in any panel.

The accuracy and the error probabilities were calculated using the above methods and the parameters in Supplementary Table S1. The results in Figure 4 are shown as histograms where the bars represent the number of positions in the operon with an error probability within that range. The ranges of error probabilities are similar to the distributions of accuracy previously published (10), and are best represented on a log scale. The error probabilities after initial selection are represented both in Figure 4A and B. In Figure 4A they are weighted by nucleotide concentration to show the error probabilities as they would appear in the model or in an experimental setup after initial selection but before proofreading. In Figure 4B, the error probabilities are not weighted by concentration, and instead represent the error probabilities after normalized initial selection discrimination, expressed in Equation (7).

The grouping of histogram data by the correct RNA nucleotide in the active site shows a clear trend that the substitution of G is strongly discriminated against in all types of selection, and that most errors appear as substitutions of C. However, when comparing panels A and B, it is seen that the poor error discrimination against substitutions of C is largely an effect of C being the least prevalent nucleotide. The strong discrimination against substitutions of G remains; however, but could be an effect of the incomplete mismatch dataset.

The error probabilities are calculated according to Equation (11), and averaged by two different methods in Table 1. First, the average log-scale error frequency is calculated as exp(mean(log(Err))). This average represents the average on the log-scale as shown in Figure 4. Second, the average error frequency per nucleotide is also calculated as mean(Err). This can be compared to the experimentally observed error frequency and is of higher biological relevance. However, due to the log-normal distribution of the error, the average error frequency is dominated by the very error-prone positions and does not give a fair representation of the error frequency spectrum.

**Table 1.** Average log-scale error and error frequency of initial selection, proofreading step 1 and 2, with and without revisiting

	Average log-scale error frequency (as exp(mean(log(Err))))	Log-scale error frequency decrease per step (as a factor)	Error frequency per nucleotide (as mean(Err))	Error frequency decrease per step (as a factor)
Initial selection	3.2·10^−3^		3.2·10^−3^	
Proofreading 1	2.5·10^−6^	1.3·10^3^	7.5·10^−5^	43
Revisiting positions, proofreading 1	1.2·10^−6^	2.1 (to Proofreading 1)	6.5·10^−5^	1.2 (to Proofreading 1)
Proofreading 1+2	1.9·10^−7^	13 (to Proofreading 1)	2.6·10^−5^	2.9 (to Proofreading 1)
Revisiting positions, Proofreading 1+2	5.1·10^−8^	3.7 (to Proofreading 2)	2.3·10^−5^	1.1 (to Proofreading 2)

The average log-scale error is calculated as exp(mean(log(Err))), meaning that it is the average on the log-scale as in Figure 4. The error frequency per nucleotide is calculated as mean(Err), meaning that it instead represents the error frequency of the transcript. The positions with very low accuracies dominate this error frequency. The contribution to the error frequency of each step is shown both cumulatively, as the total error frequency as that step is added and individually, as the factor decrease in error frequency per step.

The two averages are calculated first with only initial selection included, then with initial selection and proofreading selection step one, then with all three selection steps and finally the two latter with revisiting of positions, as shown in Table 1. Table 1 also shows the individual contributions per selection step, calculated as the factor of error frequency reduction. This is done by dividing the accuracy or error frequency of each step by that of the previous, and the error reduction factor is the inverse of the obtained number.

Figure 4 and Table 1 show that the error frequency reduction of the second proofreading step is smaller than that of the first, with an average log-scale error decrease of proofreading step 2 of 13.31 compared to 1251 for proofreading step 1. This was expected for three reasons. Firstly, the second round of proofreading has a lower maximal discrimination and accuracy than the first round since the next nucleotide addition in this elongation step is not retarded by the misincorporation. The errors in the penultimate positions are not driven toward state BACK by mismatch discrimination in the nucleotide addition cycle, so the error correction is more sensitive to the general (cognate) probability of backstepping in the second step of proofreading, which is low when the transcription speed is high.

Secondly, the effects of *F*_1_ and *F*_2_ are sensitive to the choice of parameters. With the set of parameters used here, the impaired forward reaction has a very big effect on *F*_1_, dwarfing the effect of *F*_2_, due to the dominance of the term $\frac{{{q_2}}}{{{k_{\rm 3}} \cdot [{\rm NT}{{\rm P}_{{\rm i} + 1}}]}}( {1 + \frac{{q_3^{{\rm c}/{\rm nc}}}}{{k_{\rm c}^{{\rm c}/{\rm nc}}}}} )$ in Equation (8). At increasing nucleotide concentrations, this effect is decreasing. The effect of the choice of parameters is further discussed in the Supplementary Data.

Third, the effect of the proofreading steps on the error probability of each position cannot be directly translated to the error frequency per nucleotide due to the skewness of the error probability distribution (Figure 4 and Table 1). The errors arise predominantly at positions with low accuracy, so the average number of errors in a transcript does not capture accuracy enhancements at already accurate positions. Here, *F*_1_ and *F*_2_ are weakly but significantly correlated (correlation coefficient 0.047; *P*-value 0.00052), meaning that the effect of the second step is slightly higher in the already accurate positions. Theoretically, a second selection step could increase the accuracy with almost no effect on the error frequency if it only affected high-accuracy positions. For this reason, we have chosen show both the error frequency per nucleotide and the log-scale average (Table 1).

### Effect of revisiting positions

**Figure 5.**
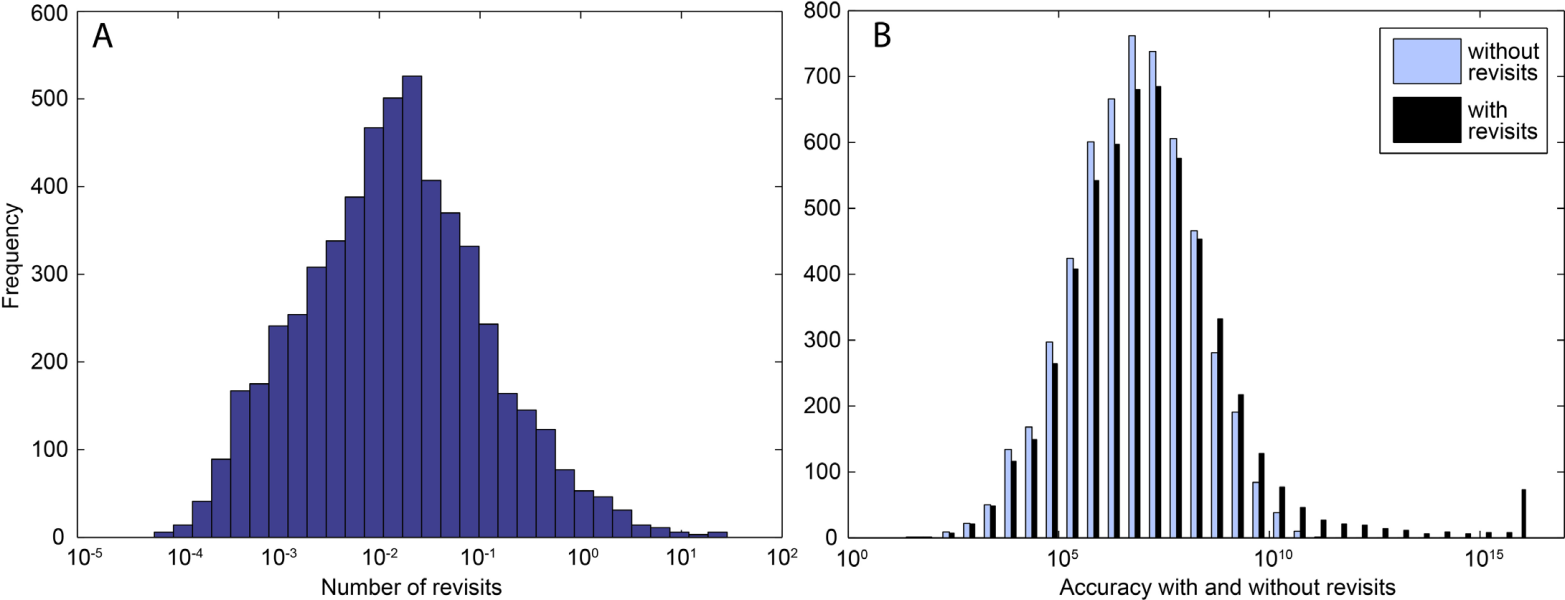
The effect of revisiting of elongation states on the template dependent total accuracy of *rrnC*. (**A**) Histogram of revisits per position. (**B**) Histograms of total accuracy with two steps of proofreading, with (black) and without (pale) accuracy amplification by revisiting positions. The total accuracy without revisiting positions is calculated by Equation (10), and the total accuracy with the effect of revisiting positions is calculated by Equation (5) in the Supplementary Data.

For the same operon, we also investigated the effect of accuracy amplification by revisiting positions, presented in Figure 5 and Table 1. The number of revisits per incorporation (Figure 5A) has a near-normal distribution on a logarithmic scale. For most positions, the expected number of revisits will be very small, typically <<1, but to a few positions the polymerase are expected to return several times, >2. The log-scale average number of revisits, calculated as exp(mean(log(*RV*))), is 0.014.

The distribution of the total accuracy amplification by revisiting positions mirrors the distribution of the revisits (Figure 5B). The effect is small over the peak of the accuracy distribution, but the accuracy amplifications give a few positions extremely high values. Error discrimination this high would have little benefit to the living cell, so these positions, or rather the positions from which the transcript is cleaved (i + 2), should probably be viewed as short pause sites rather than accuracy hotspots.

## DISCUSSION

We have presented a conceptual argument for two-step proofreading in transcript elongation based on experimental observations, and a model to predict the accuracy amplifying effect of both steps and the resultant error frequency. We note that the qualitative argument for two-step proofreading would remain unaffected even if the quantitative error model were to be significantly adjusted in the future. We also quantified the effect of the revisiting of positions. Revisiting positions is not a suggestion made by this paper but an inescapable consequence of the transcript cleavage mechanism, and we show that the benefit of revisiting positions to the error frequency is generally low.

The sequence dependence of the transcriptional accuracy model presented here is derived from varying interaction energies of pairs of base pairs as measured in solution. This is not a comprehensive description—there are other possible sources of fidelity control than the substrate–template interaction energies—which makes comparisons to experimental data all the more necessary and interesting. The strength of this description, however, is that our model takes full advantage of the accessible information, as DNA sequences are readily available. With a known genetic sequence, the model can estimate the overall transcription error rate and also suggest positions with high error probabilities.

The large sequence dependent accuracy variation of the model is evident on a nucleotide level. Comparing different genes, the result distributions and average error frequencies for the two proofreading steps are very similar. We have chosen to show only the results for *rrnC* since the parameters are tuned to its experimentally well-defined transcription time.

Measuring of the total accuracy has historically been hampered by methodological difficulties, as the method error from reverse transcriptase and DNA sequencing is usually much higher than the transcriptional error frequency. However, recent studies on transcriptional accuracy have measured the transcription error in new and interesting ways, showing promise for the future.

One of them, by Gout *et al.* (9), used a single molecule based approach to validate the true transcription errors. This gave a good estimate of the transcriptional error frequency in *Caenorhabditis elegans*, but also the sequence motifs around the errors, which allowed for accuracy prediction for the same sequences using our previous model, very similar to the one presented here but with only one proofreading step (10). A comparison showed that the model could predict a subdivision of the errors, but not all of them, just as expected from a model predicting the template-dependent accuracy variation. Nevertheless, this result verified with high statistical significance that the transcription model does explain part of the substrate selection variation in transcription (10).

The results used to verify the transcription model does not have the power to distinguish between the two proofreading steps and could not be used to test the extended model presented here. In the near future, with an accuracy landscape of base-pair resolution, we will hopefully be able to separate initial selection and the two steps of proofreading selection, by their different sequence dependence in the transcription bubble. Another way to verify the second step of proofreading could be to study the pattern of cleavage in an elongation complex with a mismatch by analyzing the cleaved-off residues, like in previous studies of transcript cleavage (41,49). In order to detect the second proofreading step, the experimental conditions must be right since the effect of the second proofreading step could otherwise be masked by the greater effect of the first step. However, the model suggests that the bigger effect of the first proofreading step could be quenched with increased nucleotide concentrations.

The total time of transcript elongation of the *rrnC* operon was calculated as described in Supplementary Data. With the present set of parameters, the total transcription time is 61.507 s with proofreading step one only and 61.515 s for two-step proofreading, meaning that the time-cost for the second step of proofreading in *rrnC* is just 0.008 s. This means that even though the increase in accuracy of the second step of proofreading is small, it comes with hardly any additional cost in transcription speed, related to previous modeling of the accuracy–speed trade-off in transcription (52). We therefore conclude that this additional proofreading step can be advantageous for the system, despite its comparably small effect. Presumably, the second step of proofreading could be more important under other conditions, like a different prokaryote with a slower growth rate. It is also possible that the second step could confer a larger benefit at certain positions with very low probabilities of backtracking in the first proofreading step, which would otherwise have very low proofreading discrimination of substrates.

Another interesting aspect of the accuracy–speed trade-off is the very wide accuracy distribution. The width of the distribution is a consequence of the great variation in free energy of formation of the transcription bubbles along the template (10). However, it seems that the polymerase invests in an unnecessarily high accuracy for the majority of sequence motifs, since most errors will arise in the left-hand tail of the accuracy distribution. The optimal strategy would be to develop a mechanism that increases the accuracy only for the error-prone positions. This is not provided in the present model either by the second step of proofreading or by the revisiting of positions, but could presumably be obtained in a model with additional discriminating reactions or mismatch-specific discrimination by the polymerase.

In our model, the accuracy variation stems from the universal physical chemistry of the interaction free energies between template and substrate. However, these are the conditions under which the polymerase and its associated factors have evolved, and the selection pressure would have been strongest in favour of those changes that reduced the most common and most critical transcription errors. It is therefore not unreasonable to assume that the polymerase effect would counteract the most common errors. Here, the free energy contributions from the polymerase are assumed to be sequence independent and uniform for simplification. This includes the general assumption that the polymerase does not affect the relation between the ground states, but also all reaction barriers, which are the same regardless of the substrate. In addition, the discrimination by the polymerase is assumed to be uniform despite indications to the contrary (41,48), due to lack of data on the polymerase effect variation. The polymerase effect is also assumed to act only on the phosphodiester bond formation and the transcript cleavage, but could also include other reactions. Considering these simplifying assumptions, and the likelihood that these interactions have evolved to remedy the error-prone positions, the sequence dependent accuracy distribution could have a different appearance. The significance of the second step of proofreading selection lies in the offer of an additional opportunity of selection evolution.

Furthermore, in our model discrimination occurs when there is a net effect of the mismatch nearest-neighbor parameter to a reaction, but it is possible that a mismatch interrupts other reactions in the nucleotide addition cycle as well, introducing more discriminating reactions to the accuracy calculation. As can be seen in Equations (8) and (9), the ratio of backward and forward translocation from state PRE has a very big effect on the proofreading selectivity. To allow for error discrimination already at these two reactions is hence a kinetic opportunity to reduce the error frequency. Specifically, it would amplify the part of the proofreading selectivity that originates in the transcript cleavage. With the present model design, the selectivity of proofreading step one is largely dictated by the discrimination in the nucleotide addition, which drives the system toward the backward reaction. Lacking this discrimination, the selectivity of proofreading step two is considerably lower. With translocation discrimination, the selectivity of both proofreading steps, but particularly of proofreading step two, would increase. Translocation discrimination has not been studied and we do not know if such discrimination exists, but given the evolutionary pressure on bacteria, it does not seem a very unreasonable scenario and was used in a previous proofreading model (45). The model of the transcriptional accuracy thus outlines the possible evolutionary adaptations to increase both accuracy and transcription speed.

## Supplementary Material

Supplementary Data
