# Supplementary material for: Transcriptional accuracy modeling suggests two-step proofreading by RNA polymerase
Source: Nucleic Acids Res. 2017 Sep 28;45(20):11582–93. doi: 10.1093/nar/gkx849 (PMC5714138; doi:10.1093/nar/gkx849)
Supplement: Supplementary Data [file gkx849_supp.pdf]

Supplemental Information:
Transcriptional accuracy modeling suggests two-step proofreading by RNA polymerase

H Mellenius and M Ehrenberg

Input parameters

The considerations of parameter choices have previously been described and are still applicable (1); however, with the new calculation of the total transcription time described below, parameters have been re-tuned here so that the total modeled transcription time of 61.5 s fits the experimental transcription time of 60 s.

In the model, the reaction rate barriers are the same for all base pairs, despite the conceivable plausibility of variations in reaction rates for different nucleotides. This has been observed for phosphodiester bond formation (2) and transcript cleavage (3), but since comprehensive data is lacking, we use only a single value.

**Parameter**	**Description**	**Value**	**Reaction rate without $\Delta\Delta G_{diff}$**	**Measured or tuned**	**Value from reference**
[ATP], [CTP], [GTP], [UTP]	*In vivo* concentrations	3560 µM ATP, 325 µM CTP, 1660 µM GTP, 667 µM UTP		Measured	The same (4)
10 DNA/DNA	Nearest-neighbor parameters	See reference		Measured	The same (5)
16 RNA/DNA	Nearest-neighbor parameters	See reference		Measured	The same (6)
32 Mismatch	Nearest-neighbor parameters	See reference		Measured	The same (7)
k_{pre}	Pre-factor	10^9 s^{-1}		From reference	10^9–10^{11} $s^{-1}M^{-1}$ for second order rate constants (8)
$k_{pre\text{-}a}$	Pre-factor of association	$5\cdot10^{13}$ s^{-1} M^{-1}		Tuned	
$\Delta G^{\ddagger}_{a}$	Reaction energy barrier of association and	$9.8\cdot R\cdot T$ $Jmol^{-1}$	$4.6\cdot10^6$ s^{-1} (association of GTP);	Tuned	

	dissociation, with rate constants k_3 and q_3		$5.5 \cdot 10^4$ s^{-1} (dissociation)		
$\Delta G^{\ddagger}_{c}$	Reaction energy barrier of phosphodiester bond formation, rate constant k_c	$13.1 \cdot R \cdot T$ $Jmol^{-1}$	$2.0 \cdot 10^3$ s^{-1}	Measured + tuned	1200 s^{-1} for RNAP II (9)
$\Delta G^{\ddagger}_{trans}$	Reaction energy barrier of translocation, with rate constants k_1, q_1, k_2 and q_2	$9.2 \cdot R \cdot T$ $Jmol^{-1}$	$1.0 \cdot 10^3$ s^{-1}	Tuned	
$\Delta G^{\ddagger}_{cut}$	Reaction energy barrier of transcript cleavage, rate constant q_c	$17.2 \cdot R \cdot T$ $Jmol^{-1}$	34 s^{-1}	Tuned	
$\Delta G^{\ddagger}_{BACK\ bias}$	Discriminating added stability to mismatched complexes in BACK	$3 \cdot R \cdot T$ $Jmol^{-1}$		Tuned	
Polymerase effect on k_c	Mismatch discrimination by the polymerase	50		Measured + tuned	3700 (at a lower polymerization rate) (2)
Polymerase effect on q_c	Mismatch discrimination by the polymerase	50		Measured + tuned	Average around 30 without cleavage factor (3)

Table S1. Summary of all input information used in the calculations. R is the gas constant, 8.314510 $J \cdot K^{-1} \cdot mol^{-1}$, and T is the temperature 310 K. The reaction rates in the fourth column are calculated without any free energy difference between ground states.

As seen in Table S1, the choice of free energy barriers for the reactions have been guided by experimental observations and measurements but not extracted directly from the references. In most cases the reference value has been measured at a lower temperature or without factors, which facilitates the measuring but gives a much slower reaction rate than the expected *in vivo* value. To some extent, this has been amended by the tuning of the parameters to the experimental total transcription time.

Association and dissociation have a common reaction barrier, $\Delta G^{\ddagger}_{a}$, but association is a faster reaction due to the association pre-factor and since the state POST·NTP is generally more stable than

POST. In addition, the pre-factor of association is tuned to a high value. Polymerase effects have been experimentally verified for both reactions (3; 2), but since the experimental conditions of the measurement were far from *in vivo* conditions, we have tuned the parameters to match experimental accuracy measurements (10; 11; 12).

The reaction rates calculated using the reaction rate barriers have been included for comparison, but without any difference in free energy between ground states. Generally, these should be the highest values as they correspond to the cases when the reactions are going from one sub-state to another with a lower free energy (and higher stability) and only the reaction rate barriers $\Delta G^{\ddagger}_{barrier}$ are used. The cases when the differences in free energy between the sub-states are also included as the reactions go to a sub-state with higher free energy would mean lower rates. The rates of nucleotide association depend on nucleotide concentration (the value shown uses the concentration of GTP), and k_c and q_c also take other values when multiplied with the polymerase discriminating effect, decreasing the rate of k_c and increasing the rate of q_c by a factor 50.

Effects of parameter choice

Figure S1 shows the error frequency distribution after two steps of proofreading but without revisiting positions for our standard parameter and five sets with altered reaction barriers for translocation (higher $\Delta G^{\ddagger}_{trans}$), association and dissociation (higher and lower $\Delta G^{\ddagger}_{a}$), phosphodiester bond formation (higher $\Delta G^{\ddagger}_{c}$) and transcript cleavage (lower $\Delta G^{\ddagger}_{cut}$). The barriers are changed by an added or subtracted 2RT (translating to a reaction rate change of a factor 7.4), but the total error distributions are similar. There are many other combinations of parameter changes that we could have chosen to show, especially since the effects of the parameters interact, so this figure does not explore the entire parameter space but gives a brief overview.

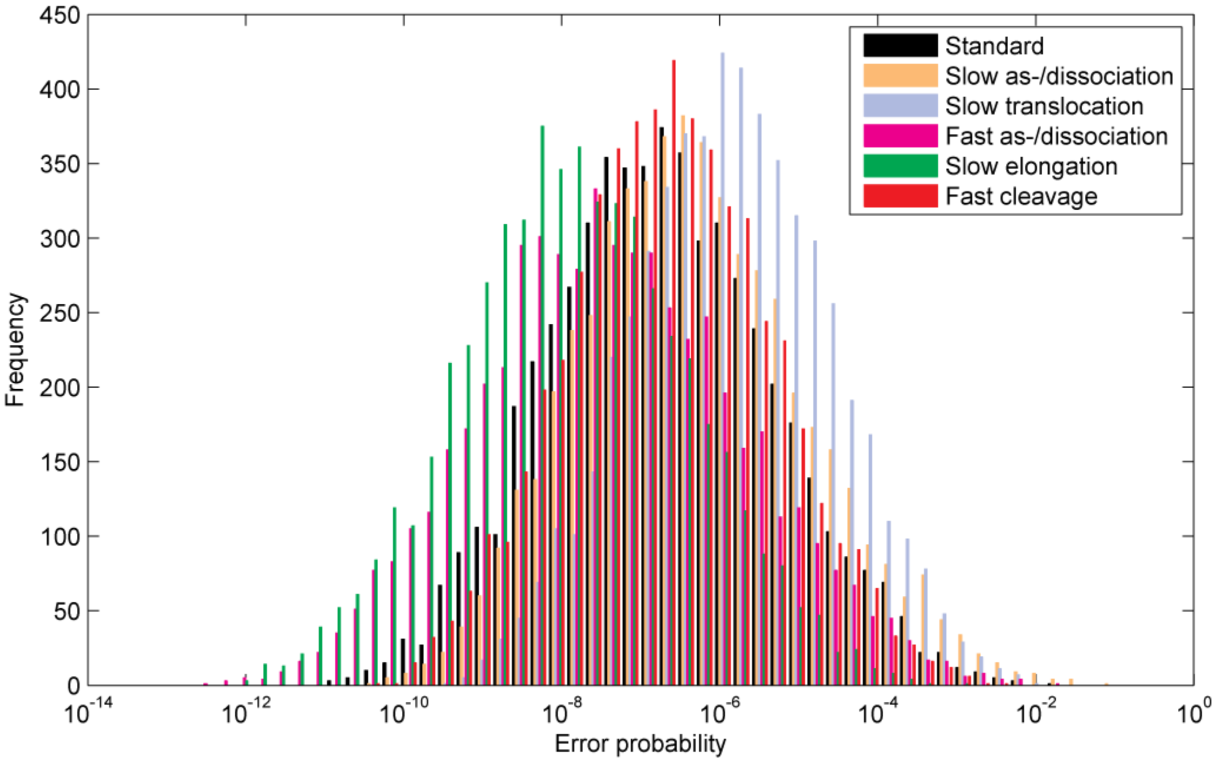

Figure S1. Error probability histograms for a few parameter sets. Black: the standard set as presented in Table S1. Beige: like the black one but $\Delta G^{\ddagger}_{trans}$ is increased from 9.2 to 11.2, so that the rate of translocation is decreased. Blue: like the black

one but $\Delta G^{\ddagger}_{a}$ is decreased from 9.8 to 7.8, so that the rates of nucleotide association and dissociation are increased. Magenta: like the black one but $\Delta G^{\ddagger}_{a}$ is increased from 9.8 to 11.8, so that the rates of nucleotide association and dissociation are decreased. Green: like the black one but $\Delta G^{\ddagger}_{c}$ is increased from 13.1 to 15.1, so that the rate of phosphodiester bond formation is decreased. Red: like the black one but $\Delta\Delta G_{cut}$ is decreased from 17.2 to 15.2, so that the rate of transcript cleavage is increased.

Table S2 complements Fig. S1 by showing the transit times, ratio of first and second step proofreading selection (mean(F_1)/mean(F_2)) and the revisiting effect to second step of proofreading as a factor of the error frequency decrease for the same parameter sets. This is a further demonstration of how the parameter dependence of the model is complex and not easy to predict. From Table S2, we see that the transit time and the relation between the two steps of proofreading vary considerably, but the effect of revisiting positions is always small. It also demonstrates that the chosen parameters were tuned to obtain a transit time close to the experimental transit time of roughly 60 s.

**Parameter set**	**Parameter different from standard**	**Transit time**	**Mean(F_1)/mean(F_2)**	**Revisiting effect to error frequency after 2-step proofreading**
Standard	(as described in Table S1)	61.5 s	231.7	1.11
Slow translocation	$\Delta G^{\ddagger}_{trans}$ = 11.2	5086.1 s	37.57	1.33
Fast as-/dissociation	$\Delta G^{\ddagger}_{a}$ = 7.8	52.3 s	240.1	1.10
Slow as-/dissociation	$\Delta G^{\ddagger}_{a}$ = 11.8	202.6 s	185.2	1.18
Slow elongation	$\Delta G^{\ddagger}_{c}$ = 15.1	406.5 s	1022.4	1.30
Fast cleavage	$\Delta G^{\ddagger}_{cut}$ = 15.2	2107.4 s	232.4	1.60

Table S2. Transit time, F_1/F_2 ratio and revisit effect for the alternative parameter sets shown in Fig. S1.

Revisiting positions

There are two fundamental differences between arriving at an elongation state by transcript elongation or by transcript cleavage. After elongation, the last nucleotide in the transcript has just undergone initial selection and is checked by proofreading for the first time. When instead returning to a position by cleavage of the nucleotides ahead, the last two nucleotides of the transcript in the state will undergo an additional round of proofreading, corresponding to proofreading step two for the penultimate nucleotide, and proofreading step one followed by proofreading step two for the last nucleotide. The accuracy of the respective rounds of proofreading is not affected, but the total accuracy is amplified by the number of “extra” visits to a position. Consequently, we have to consider how often this occurs when determining the sequence effect on proofreading selection.

The dynamics of the elongation states along the RNA is expressed by a system of master equations. The reactions connecting the elongation states, comprising the sub-state reactions, are elongation at a compound rate constant κ and transcript cleavage at a compound rate constant ς, as described in the main text (Fig. 1A) and previously (1). The other fundamental difference between arriving at an elongation state by transcript elongation or by transcript cleavage is the sub-state; directly after cleavage the polymerase is in the sub-state POST instead of PRE, the first sub-state after phosphodiester bond formation. Starting in state POST, κ and ς will not be the same as when starting in state PRE. Consequently, the ratio ς/κ in proofreading selection is also different, so that the proofreading selections F_Q for the first and second proofreading step on returning to a position (Eq. 1 and 2) differs from F, the proofreading steps after initial selection described in the main text (main text Eq. 8 and 9), here referred to as F_K.

$$F_{1Q}=\frac{P^{c}_{F1Q}}{P^{nc}_{F1Q}}=\frac{1+\left(\frac{\varsigma}{\kappa}\right)^{nc}_{1Q}}{1+\left(\frac{\varsigma}{\kappa}\right)^{c}_{1Q}};\quad \left(\frac{\varsigma}{\kappa}\right)^{c/nc}_{1Q}=\frac{q_2}{k_3\cdot[NTP_{i+1}]}\frac{1+\frac{q_3^{c/nc}}{k_c^{c/nc}}}{1+\frac{k_2}{k_1^{c/nc}}\left(1+\frac{q_1^{c/nc}}{q_c^{c/nc}}\right)} \tag{1}$$

Equivalently, for the second proofreading step:

$$F_{2Q}=\frac{P^{c}_{F2Q}}{P^{nc}_{F2Q}}=\frac{1+\left(\frac{\varsigma}{\kappa}\right)^{nc}_{2Q}}{1+\left(\frac{\varsigma}{\kappa}\right)^{c}_{2Q}};\quad \left(\frac{\varsigma}{\kappa}\right)^{c/nc}_{2Q}=\frac{q_2}{k_3\cdot[NTP_{i+1}]}\frac{1+\frac{q_3}{k_c}}{1+\frac{k_2}{k_1^{c/nc}}\left(1+\frac{q_1^{c/nc}}{q_c^{c/nc}}\right)} \tag{2}$$

Therefore, we have designed a master equation where each elongation state E_i is represented twice; both when reached by the forward reaction (denoted by subscript *K*) and by the backward reaction (denoted by subscript *Q*):

$$\begin{aligned}\frac{dP(E_{K(i)})}{dt}&=-\left(\kappa_{K(i)}+\varsigma_{K(i)}\right)\cdot P(E_{K(i)})+\kappa_{K(i-1)}\cdot P(E_{K(i-1)})+\kappa_{Q(i-1)}\cdot P(E_{Q(i-1)})\\ \frac{dP(E_{Q(i)})}{dt}&=-\left(\kappa_{Q(i)}+\varsigma_{Q(i)}\right)\cdot P(E_{Q(i)})+\varsigma_{K(i+2)}\cdot P(E_{K(i+2)})+\varsigma_{Q(i+2)}\cdot P(E_{Q(i+2)})\end{aligned} \tag{3}$$

The relations between the double elongation states are represented in Fig. S2, and described further below in the calculations of the total transcription time.

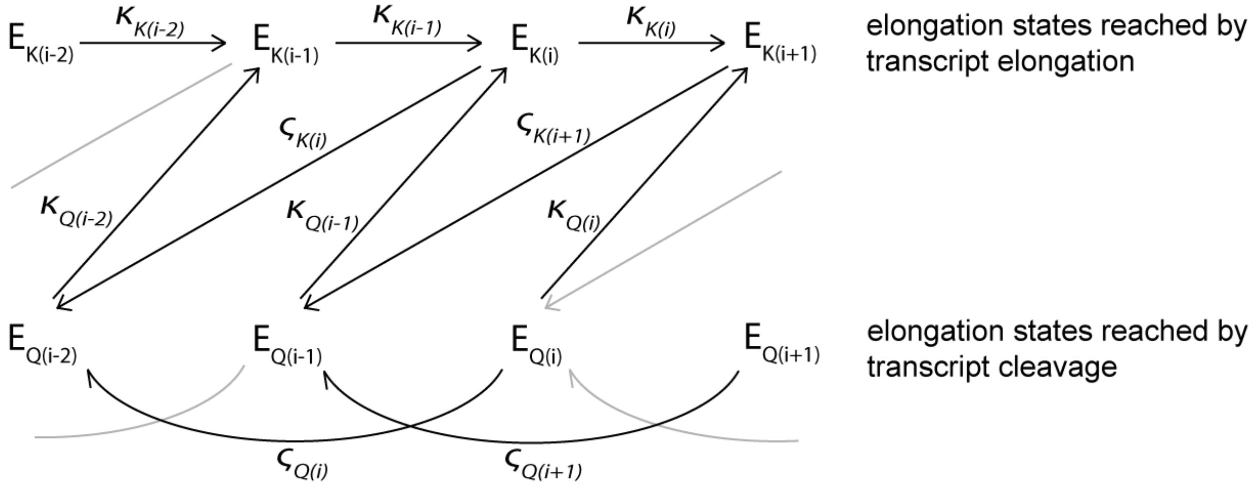

Figure S2. The reaction scheme of the double elongation states.

The boundary conditions, that the system starts at state PRE in the first position in the operon from which it cannot backtrack and finishes when it reaches the termination site, allow us to calculate the mean time spent in each elongation state (1). The mean time spent in each elongation state divided by the mean time to leave it gives us the mean number of times each state is visited, and thus we know how often each position is revisited. The number of extra rounds of proofreading (RV_i) is the

number of revisits before the nucleotide is cleaved off or transcription is terminated, which equals the number of revisits per incorporations:

$$\text{Revisits per incorporated nucleotide} = \frac{\text{Number of visits in backward states}}{\text{Number of visits in forward states}} = RV \quad (4)$$

The probability of product formation for an incoming nucleotide at position *i* equals the probability to go through initial selection, proofreading selection step one, proofreading selection step one after returning to the position *i* a total of RV_i number of times, proofreading selection step two after step one ($1+RV_i$) number of times, and proofreading selection step two after returning to the position *i+1* a total of RV_{i+1} number of times. Only if the substrate escapes rejection in all these steps, with the respective probabilities P_I, P_{F1K}, P_{F1Q}^{RV}, P_{F2K}^{1+RV} and $P_{F2Q}^{RV_{i+1}}$, will it persist until transcript termination.

In analogy with the main text Eq. 10, the total accuracy per non-cognate substrate and nucleotide position based on this formulation of probability of product formation becomes:

$$A_{tot,i}\left(\left[S\right]\right) = \frac{k_a\left[S_i^c\right]}{k_a\left[S_i^{nc}\right]} \cdot I_i \cdot F_{1i} \cdot F_{2i} = \frac{\left[S_i^c\right]}{\left[S_i^{nc}\right]}\left(\frac{P_I^c}{P_I^{nc}}\right)_i\left(\frac{P_{F1K}^c}{P_{F1K}^{nc}}\right)_i\left(\frac{P_{F1Q}^c}{P_{F1Q}^{nc}}\right)_i^{RV_i}\left(\frac{P_{F2K}^c}{P_{F2K}^{nc}}\right)_i^{1+RV_i}\left(\frac{P_{F2Q}^c}{P_{F2Q}^{nc}}\right)_i^{1+RV_i} \quad (5)$$

In Eq. 5, RV_{i+1} is the number of revisits to position *i+1*. Since the misincorporation does not affect the transcription bubble stability after leaving the active site by polymerase translocation, the mean number of returns RV_i or RV_{i+1} will not depend on the identity of the incorporated nucleotide, only on the position *i*. The other variables in Eq. 5 are specific both for the position and the incorporated substrate.

We make the assumption that the rest of the transcription bubble, aside from the scrutinized position, is always correct. This means that the possible effect on the transcription bubble stability of the misincorporated base when it exits the polymerase after eight elongations is neglected. The probabilities of misincorporations in the rest of the transcript are, however, included in the reaction rate constants between elongation states (see below) so that the number of revisits includes the increased chance of cleavage of mismatches.

The model still only allows for one step of backtracking even though long backtracking should be included in a model of the total backtracking dynamics. It is, however, not important for the purpose of analyzing the accuracy effect. Since the accuracy enhancement comes from the number of cleavages to a certain position, it is the total number of cleavages and not the length of the transcript cleaved off that determines the accuracy enhancement. For this reason, the effect on accuracy of long backtracking would probably be small and the effect on proofreading selection of revisiting positions does not motivate its inclusion.

Calculation total transcription time

The total transcription time was previously (1) calculated by numeric integration of the master equation describing the model. The master equation is the time derivative of the probability $P(E_i)$ of being in each of the elongation states, given by the compound rate constants of going to and from the states (Eq. 6). The integrals of the equations in the master equation express the mean times τ_i of which each elongation state is inhabited by the polymerase. The boundary conditions are that the

polymerase starts at the start of transcription, and finishes at the termination site at length *N*. It cannot go backwards from the first two positions, and the two last positions can only be reached by forward reactions.

$$
\begin{aligned}
&\frac{dP(E_1)}{dt} = -\kappa_1 \cdot P(E_1) + \varsigma_3 \cdot P(E_3) \Rightarrow \\
&-1 = -\kappa_1 \cdot \tau_1 + \varsigma_3 \cdot \tau_3 \\
&\frac{dP(E_2)}{dt} = -\kappa_2 \cdot P(E_2) + \kappa_1 \cdot P(E_1) + \varsigma_4 \cdot P(E_4) \Rightarrow \\
&0 = -\kappa_2 \cdot \tau_2 + \kappa_1 \cdot \tau_1 + \varsigma_4 \cdot \tau_4 \\
&\frac{dP(E_i)}{dt} = -(\kappa_i + \varsigma_i) \cdot P(E_i) + \kappa_{i-1} \cdot P(E_{i-1}) + \varsigma_{i+2} \cdot P(E_{i+2}) \Rightarrow \\
&0 = -(\kappa_i + \varsigma_i) \cdot \tau_i + \kappa_{i-1} \cdot \tau_{i-1} + \varsigma_{i+2} \cdot \tau_{i+2} \\
&\frac{dP(E_{N-1})}{dt} = -(\kappa_{N-1} + \varsigma_{N-1}) \cdot P(E_{N-1}) + \kappa_{N-2} \cdot P(E_{N-2}) \Rightarrow \\
&0 = -(\kappa_{N-1} + \varsigma_{N-1}) \cdot \tau_{N-1} + \kappa_{N-2} \cdot \tau_{N-2} \\
&\frac{dP(E_N)}{dt} = -(\kappa_N + \varsigma_N) \cdot P(E_N) + \kappa_{N-1} \cdot P(E_{N-1}) \Rightarrow \\
&0 = -(\kappa_N + \varsigma_N) \cdot \tau_N + \kappa_{N-1} \cdot \tau_{N-1}
\end{aligned}
\tag{6}
$$

However, in this study we extended this set of equation to the double equations including the states reached by cleavage, since the compound reaction rate constants κ and ς will be different when the polymerase starts in sub-state POST instead of PRE. Each elongation state in Eq. 6 is instead described by two probabilities in the master equation, representing the probabilities of being in elongation state *i* after a forward reaction ($P(E_{K(i)})$) or a backward reaction ($P(E_{Q(i)})$):

$$
\begin{aligned}
\frac{dP(E_{K(i)})}{dt} &= -\left(\kappa_{K(i)} + \varsigma_{K(i)}\right) \cdot P(E_{K(i)}) + \kappa_{K(i-1)} \cdot P(E_{K(i-1)}) + \kappa_{Q(i-1)} \cdot P(E_{Q(i-1)}) \\
\frac{dP(E_{Q(i)})}{dt} &= -\left(\kappa_{Q(i)} + \varsigma_{Q(i)}\right) \cdot P(E_{Q(i)}) + \varsigma_{K(i+2)} \cdot P(E_{K(i+2)}) + \varsigma_{Q(i+2)} \cdot P(E_{Q(i+2)})
\end{aligned}
\tag{7}
$$

Here, $\kappa_{K(i)}$ is the rate constant of leaving a state reached by elongation (to sub-state PRE) by another elongation; $\kappa_{Q(i)}$ is the rate constant of leaving a state reached by cleavage (to sub-state POST) by transcript elongation; $\varsigma_{K(i)}$ is the rate constant of leaving a state reached by elongation (to sub-state PRE) by transcript cleavage and $\varsigma_{Q(i)}$ is the rate constant of leaving a state reached by cleavage (to sub-state POST) by transcript cleavage.

Solving the equation system of the integrated double master equation gives us the mean dwelling times of both forward and backward elongation states, and summation of all elongation states gives us the total transit time of the transcript.

To do this, however, we must solve another set of master equations; the master equation of the sub-states needs to be solved for each elongation state. The elongation reaction rate constants κ and ς at which the polymerase moves from one elongation state to another, are calculated from the reaction rate constants k_c and q_c of the phosphodiester bond formation and transcript cleavage, respectively,

multiplied by the probability of being in that sub-state. The probability of being in the sub-state equals the fraction of the mean time to leave the elongation state that this sub-state is inhabited, so that:

$$\kappa = k_c \frac{\tau_{\text{POST}\cdot\text{NTP}}}{\tau_{\text{BACK}} + \tau_{\text{PRE}} + \tau_{\text{POST}} + \tau_{\text{POST}\cdot\text{NTP}}}, \ \varsigma = q_c \frac{\tau_{\text{BACK}}}{\tau_{\text{BACK}} + \tau_{\text{PRE}} + \tau_{\text{POST}} + \tau_{\text{POST}\cdot\text{NTP}}} \tag{8}$$

Note that the time τ_i is the total (mean) time spent in the elongation state, and does not equal the total time to leave it, which is the sum of the mean times of the sub-states. The time τ_i also reflects the relation between the elongation state and its neighbors, taking into account that the state might be visited more than once. The ratio between the total time and the mean time to leave the state gives us the mean number of visits, as mentioned in the main text.

The mean times of the sub-states are described by the integrated master equation:

$$\begin{aligned}
&\frac{dP_{\text{PRE}}}{dt} = -(k_2 + k_1)\cdot P_{\text{PRE}} + q_1 \cdot P_{\text{BACK}} + q_2 \cdot P_{\text{POST}} \Rightarrow \\
&-1 \text{ or } 0 = -(k_2 + k_1)\cdot \tau_{\text{PRE}} + q_1 \cdot \tau_{\text{BACK}} + q_2 \cdot \tau_{\text{POST}} \\
&\frac{dP_{\text{POST}}}{dt} = -(q_2 + k_3)\cdot P_{\text{POST}} + k_2 \cdot P_{\text{PRE}} + q_3 \cdot P_{\text{POST}\cdot\text{NTP}} \Rightarrow \\
&0 \text{ or } -1 = -(q_2 + k_3)\cdot \tau_{\text{POST}} + k_2 \cdot \tau_{\text{PRE}} + q_3 \cdot \tau_{\text{POST}\cdot\text{NTP}} \\
&\frac{dP_{\text{BACK}}}{dt} = -(q_1 + q_c)\cdot P_{\text{BACK}} + k_1 \cdot P_{\text{PRE}} \Rightarrow \\
&0 = -(q_1 + q_c)\cdot \tau_{\text{BACK}} + k_1 \cdot \tau_{\text{PRE}} \\
&\frac{dP_{\text{POST}\cdot\text{NTP}}}{dt} = -(q_3 + k_c)\cdot P_{\text{POST}\cdot\text{NTP}} + k_3 \cdot P_{\text{POST}} \Rightarrow \\
&0 = -(q_3 + k_c)\cdot \tau_{\text{POST}\cdot\text{NTP}} + k_3 \cdot \tau_{\text{POST}}
\end{aligned} \tag{9}$$

There are two sets of boundary conditions that give two different solutions; for the elongation states reached by transcript elongation, the system of sub-states starts in state PRE, so that the solution to the integral of the time-derivate of the probability of being in state PRE is -1, and for elongation states reached by transcript cleavage, the system of sub-states starts in POST. The two solutions give the two different values for κ and ς; $\kappa_{K(i)}$ and $\varsigma_{K(i)}$, and $\kappa_{Q(i)}$ and $\varsigma_{Q(i)}$; and they in turn give the two different solutions to ς/κ that constitute the difference between F_{1K} and F_{1Q}, as well as F_{2K} and F_{2Q} (Eq. 8 and 9 in the main text and Eq. 1 and 2 here).

There is one more adjustment to the calculation of the total transcription time; including the errors. As we know, the reaction rate constants differ for cognate and non-cognate substrates. Every time an elongation state is visited, both for the first time and when revisited, there is a chance that the last or second-to-last incorporated substrate is a mismatch. Therefore, the reaction rate constants used in the transcription time calculations are a composite of the cognate and non-cognate reaction rates, each weighted by the average error probability.

The average error probability, equal to the per-position error frequency, is estimated differently for different cases. In a forward elongation state, reached by transcript elongation, the error probability is the error rate after initial selection in the case with only one-step proofreading. This must be true

since the last incorporated nucleotide has not yet undergone proofreading and since errors in the penultimate position of the transcript are not detected. The error rate after initial selection is hence:

$$\text{Error rate after initial selection } = \frac{1}{1+I} \tag{10}$$

In a backward elongation state, reached by transcript cleavage, the error probability is the error rate after initial selection, one round of proofreading in a forward state (F_{1K}) and an average number of rounds of proofreading in a revisited state (F_{1Q}), in the case with only one-step proofreading. This average number of rounds of proofreading equals half the number of total revisits to the position, so that the error rate becomes:

$$\text{Error rate in revisited state } i\text{, 1-step proofreading} = \frac{1}{1+I\cdot F_{1K}\cdot F_{1Q}^{RV_i/2}} \tag{11}$$

With the two-step proofreading, errors in the penultimate position are also detected. In a forward state, reached by elongation, the average error is the error rate at the last position, after initial selection, plus the estimated error rate at the penultimate position when the elongation state after the next is reached by elongation. An error in the penultimate position must have gone through initial selection and one round of step-one proofreading after elongation, but it is also possible that the polymerase has revisited the state of the step-one proofreading, in which case this nucleotide has also gone through step-two proofreading in a forward state. Lastly, it is also possible that this position has been revisited, whereby the penultimate nucleotide has undergone step-two proofreading in the revisited state. Hence, the error rate in the penultimate position must account for the chance of an error persisting initial selection, one round of step-one proofreading in a forward state (F_{1K}), an average number of rounds of step-one proofreading in a revisited state (F_{1Q}), an average number of rounds of step-two proofreading in the forward state (F_{2K}), and an average number of rounds of step-two proofreading in a revisited state (F_{2Q}). The average number of rounds of step-two proofreading in a forward state is estimated as half the number of revisits to the preceding position, so that:

$$\text{Error rate in penultimate position } i\text{, forward state } = \frac{1}{1+I\cdot F_{1K}\cdot F_{1Q}^{RV_i/2}\cdot F_{2K}^{RV_i/2}\cdot F_{2Q}^{RV_{i+1}/2}} \tag{12}$$

Lastly, in a revisited state, reached by transcript cleavage, the average error again is the error rate at the last position, after initial selection, plus the estimated error rate at the penultimate position when the elongation state after the next is reached by elongation. However, when the position is revisited we know for certain that the error in the penultimate position has passed through step-two proofreading in the forward state (F_{2K}) at least once. Hence, the error rate becomes:

$$\text{Error rate in penultimate position } i\text{, revisited state } = \frac{1}{1+I\cdot F_{1K}\cdot F_{1Q}^{RV_i/2}\cdot F_{2K}^{1+RV_i/2}\cdot F_{2Q}^{RV_{i+1}/2}} \tag{13}$$

This can be compared to the total accuracy after the full number of revisits (Eq. 5).

The composite reaction rate constant for an arbitrary reaction between elongation states hence becomes:

$$\kappa = \kappa^{c}(1 - P_{error}) + \kappa^{nc} \cdot P_{error} \quad \text{or} \quad \varsigma = \varsigma^{c}(1 - P_{error}) + \varsigma^{nc} \cdot P_{error} \tag{14}$$

The attentive reader will have realized that not only are the error probabilities calculated using the number of revisits, *RV*, but also that the number of revisits is calculated using the error probabilities. This self-reference is solved by iterating the calculation of revisits until the solution of the total transcription time is stable; for most parameter sets, this is reached in no more than three cycles, starting with the solution for revisits with cognate reaction rates only.

Through these somewhat cumbersome calculations, the total time of transcript elongation for the whole operon is estimated both for the model with only one step of proofreading and for the two-step proofreading. Compared to the calculation used in the previously published model (1), without non-cognate reaction rates and the double elongation states, the total transcription time increased by around 50%, motivating re-tuning of the parameters to match the experimental transcription rate. This increase was mostly due to the non-cognate reaction rate constants.

Supporting References

1. Mellenius,H. and Ehrenberg,M. (2015) DNA template dependent accuracy variation of nucleotide selection in transcription. PLoS ONE, 10, e0119588.
2. Yuzenkova,Y., Bochkareva,A., Tadigotla,V.R., Roghanian,M., Zorov,S., Severinov,K. and Zenkin,N. (2010) Stepwise mechanism for transcription fidelity. BMC Biol., 54, 1-15.
3. Zenkin,N., Yuzenkova,Y. ad Severinov,K. (2006) Transcript-assisted transcriptional proofreading. Science, 313, 518-520.
4. Buckstein,M.H., He,J. and Rubin,H. (2008) Characterization of nucleotide pools as a function of physiological state in Escherichia coli. J. Bacteriol., 190, 718-726.
5. SantaLucia,J. (1998) A unified view of polymer, dumbbell, and oligonucleotide DNA nearest-neighbor thermodynamics. Proc. Natl. Acad. Sci. U. S. A., 95, 1460-1465.
6. Sugimoto,N., Nakano,S., Katoh,M., Matsumura,A., Nakamuta,H., Ohmichi,T., Yoneyama,M. and Sasaki,M. (1995) Thermodynamic parameters to predict stability of RNA/DNA hybrid duplexes. Biochemistry, 34, 11211-11216.
7. Watkins,N.E., Kennelly,W.J., Tsay,M.J., Tuin,A., Swenson,L., Lee,H.R., Morosyuk,S., Hicks,D.A. and SantaLucia,J. (2011) Thermodynamic contributions of single internal rAdA, rCdC, rGdG and rUdT mismatches in RNA/DNA duplexes. Nucleic Acids Res., 39, 1894-1902.
8. Fersht,A. (1999) Structure and mechanism in protein science: A guide to enzyme catalysis and protein folding. W.H. Freeman and Company, N.Y., pp. 158-163.
9. Nedialkov,Y.A., Gong,X.Q., Hovde,S.L., Yamaguchi,Y., Handa,H., Geiger,J.H., Yan,H. and Burton,Z.F. (2003) NTP-driven translocation by human RNA polymerase II. J. Biol. Chem., 278, 18303-18312.
10. Ninio,J. (1991) Connections between translation, transcription and replication error-rates. Biochimie, 73, 1517-1523.
11. Imashimizu,M., Oshima,T., Lubkowska,L. and Kashlev,M. (2013) Direct assessment of transcription fidelity by high-resolution RNA sequencing. Nucleic Acids Res., 41, 9090-9104.
12. Gout,J.F., Thomas,W.K., Smith,Z., Okamoto,K. and Lynch,M. (2013) Large-scale detection of in vivo transcription errors. Proc. Natl. Acad. Sci. U. S. A., 110, 18584-18589.
